# Automation of Article Selection Process in Systematic Reviews Through Artificial Neural Network Modeling and Machine Learning: Protocol for an Article Selection Model

**DOI:** 10.2196/26448

**Published:** 2021-06-15

**Authors:** Gabriel Ferraz Ferreira, Marcos Gonçalves Quiles, Tiago Santana Nazaré, Solange Oliveira Rezende, Marcelo Demarzo

**Affiliations:** 1 Department of Science and Technology Universidade Federal de São Paulo São Paulo Brazil; 2 Universidade de São Paulo São Paulo Brazil

**Keywords:** deep learning, machine learning, systematic review, mindfulness

## Abstract

**Background:**

A systematic review can be defined as a summary of the evidence found in the literature via a systematic search in the available scientific databases. One of the steps involved is article selection, which is typically a laborious task. Machine learning and artificial intelligence can be important tools in automating this step, thus aiding researchers.

**Objective:**

The aim of this study is to create models based on an artificial neural network system to automate the article selection process in systematic reviews related to “Mindfulness and Health Promotion.”

**Methods:**

The study will be performed using Python programming software. The system will consist of six main steps: (1) data import, (2) exclusion of duplicates, (3) exclusion of non-articles, (4) article reading and model creation using artificial neural network, (5) comparison of the models, and (6) system sharing. We will choose the 10 most relevant systematic reviews published in the fields of “Mindfulness and Health Promotion” and “Orthopedics” (control group) to serve as a test of the effectiveness of the article selection.

**Results:**

Data collection will begin in July 2021, with completion scheduled for December 2021, and final publication available in March 2022.

**Conclusions:**

An automated system with a modifiable sensitivity will be created to select scientific articles in systematic review that can be expanded to various fields. We will disseminate our results and models through the “Observatory of Evidence” in public health, an open and online platform that will assist researchers in systematic reviews.

**International Registered Report Identifier (IRRID):**

PRR1-10.2196/26448

## Introduction

### Background

A systematic review (SR) [1] can be defined as a summary of the evidence found in the literature. Unlike classic reviews, specific and described methods are used in the literature search to reach certain results using scientific articles [2]. Essentially, a systematic review consists of a thorough and extensive survey of all published studies on a topic combined with a thorough analysis of the results. In addition, an SR is an extensive systematized survey on a specific subject in which a careful analysis of the evaluated outcomes is performed in an attempt to reach a single conclusion based on all included studies [2]. Reviews are increasingly common; it is estimated that 11 new reviews have been published per day since 2010 [3].

The SR should be conducted considering the factors of Population, Intervention, Control, and Outcome (PICO) [4]. In some cases, it is possible to combine the data in the articles included in an SR and perform a group analysis. This is called the statistical meta-analysis method, in which the variables that are common through similar outcomes can be analyzed in group order to synthesize the effect seen in the studies [5,6]. A meta-analysis is a mathematical calculation that combines the results of several related studies. There are cases in which it is not possible to conduct a meta-analysis, so an SR would be limited to qualitative comparisons [7].

In addition to the systematized search, specific criteria are used to evaluate the methodological quality of each selected article. These measurements are made using scores that vary according to the design of the study in question [8-10]. The search is conducted in various databases to reach the largest number of scientific articles and reduce the risk of failing to include a study that could potentially affect the final result. Thus, once the studies are combined, potential articles that have not been identified are searched in the so-called gray literature.

The literature search using the chosen keywords should be described such that any other researcher could reproduce the same results as the authors, proving that the search was systematized, and the included articles were not preselected, avoiding a selection bias in the final result. However, after combining the results found in the databases, the article selection process according to the PICO criteria can be laborious and time consuming.

According to the PRISMA (Preferred Reporting Items for Systematic Reviews and Meta-Analyses) guidelines [4], the recommended approach is for two investigators to perform the same search and compare the final result. If there is disagreement regarding the selected studies, the senior investigator should make the final decision. The selection is made by reading the titles and abstracts, excluding irrelevant articles, duplicates, letters to the editor, reviews, and other publication types.

When researchers start an SR, most of the time they find a large volume of data, requiring time for a selection of articles based on their design and theme. Researchers have estimated that a systematic review can take 1046 hours or up to 26 weeks to perform [11].

Thus, to speed up the article selection process, it is possible to automate this process through a semiautomatic computer system that contains a machine learning method. The topic is broad, but if we were to restrict the idea to searching and learning with written words, the ideal model for automating article selection is artificial neural networks (ANNs) [12].

Similar to the biological model, ANNs are networks formed by several interconnected units. These connections are associated with synaptic weights that are responsible for learning on the network. The learning capacity of the network is directly linked to the number of neurons and connections. Even formed by simple units, ANNs intelligence emerges from its (network) connectivity.

One of the future goals of ANNs is to replace manual design features, which were believed to be subjective, via efficient algorithms for learning and decision making without continuous human guidance.

There are some published studies that have tested the use of artificial intelligence to improve systematic reviews. A system created by Cochrane with machine leaning algorithms enabled the identification of randomized clinical trials [13], just as Cohen and collaborators [14] launched a tool to estimate this probability from PubMed articles.

Other published articles attempted to automate the selection of studies in systematic reviews. Wallace et al [15] developed Abstrackr based on an active learning system (Active Learning) and used PubMed as a database, unlike our project that will cover other databases, using ANN. Another platform, RobotAnalyst, was created by Przybyła et al [16], but it presents a search and use methodology for artificial intelligence different from this study.

An article reading and selection system for SRs must be specialized to each field of interest and consider all selection stages. Generic software may not be sufficiently sensitive to achieve the accuracy of results afforded by using a complex tool.

### Objective

The objective of this study is to develop a semiautomatic, dynamic, and open source computer system which will carry out the selection of scientific articles in SR, specifically in the area of “Mindfulness and Health Promotion,” after deleting duplicate articles and cleaning the data.

## Methods

### Overview

The study was approved by the local ethics committee (Federal University of São Paulo – Number 9425030220-2020). The project will be conducted using Python programming software and packages. Both Python and packages are freely available online.

The system will be constructed based on the search and selection structure of the 10 most cited SRs in Scopus in two different fields: “Mindfulness and Health Promotion” and “Orthopedics” (control group), a specific area of knowledge for the authors. We will extract the PICO elements from each published SR and reperform the search in PubMed, Web of Science, and Embase. Next, articles will be manually selected for use as a template and metric for comparing the results.

**Figure 1:**
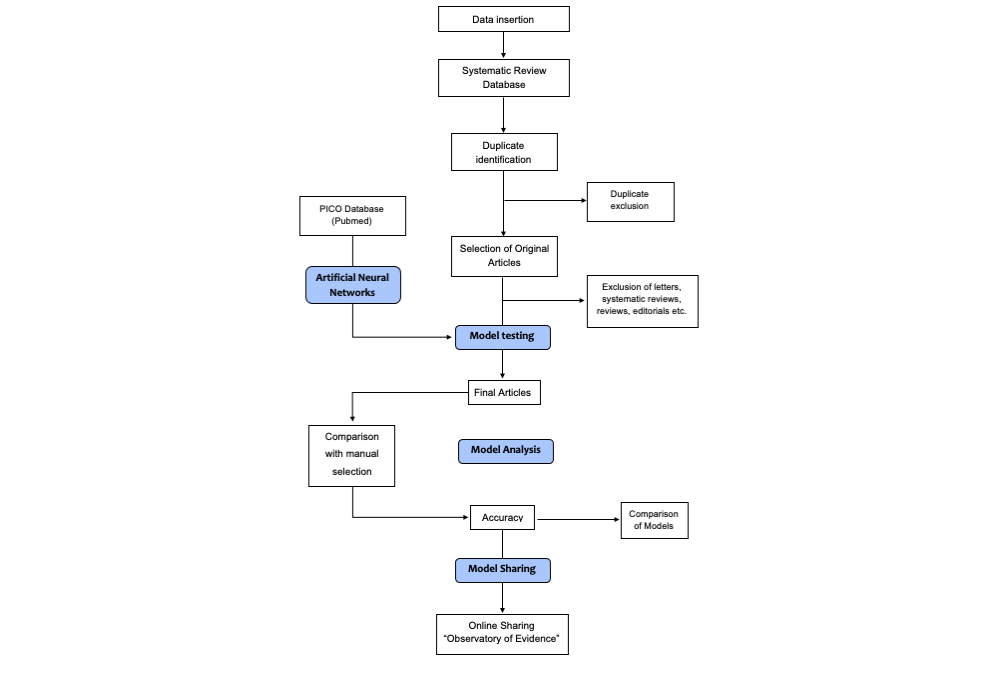
Flowchart of the automated steps in the article selection system for systematic reviews. PICO: Population, Intervention, Control, and Outcome.

A database will be created with the returned search results for each SR. The search results will be individually introduced into the selection system. Each published SR will have its own specific database and will be run in the system. Next, comparative analyses will be performed within the same field (intergroup comparison) and between two different fields (intragroup comparison). The system flowchart is shown in Figure 1.

The system will consist of six main steps: (1) data import, (2) exclusion of duplicates, (3) exclusion of nonarticles, (4) article reading and model development in ANN, (5) comparison of models, and (6) system sharing. The ANN will only be used in the fourth step. Search models simpler than the ANN will be used in other steps to prevent data loading and processing from slowing down and destabilizing the system.

The steps will be performed for each of the SR databases included in this study (for a total of 20 reviews). The steps used to implement this protocol are described in the following sections.

### Data Import

Data will be imported for each search return of the SRs. The data will be imported directly from the database of the research platforms and converted into a data frame. We will use all available data: title, abstract, year, authors, journal, and other information.

### Exclusion of Duplicates

The data must be cleaned before the articles found in the search are read. In this step, we will exclude all types of duplicate documents. We will use the digital object identifier for initial filtering and exclusion. Subsequently, the title and abstract of the articles will be compared to reduce the risk of nonidentification.

### Exclusion of Nonarticles

After the exclusion of duplicates, the data must be cleaned to exclude documents types found in the search that are not relevant to the required format and, at this time, the content. These document types include, among others, editorials, comments, author responses, reviews, and systematic reviews.

We have chosen not to build a neural network model, which would make the system slow and cumbersome. We consider that all these document types can be located and excluded through the search for regular expressions. Conducting manual article selection in parallel will provide a list of regular expressions related to each irrelevant document type to add to an SR.

### Article Reading and Model Development in ANN

Learning the documents returned from the search databases will yield only articles potentially includible in the SRs. The article titles and abstracts will be read during the manual search.

The objective is to find studies that meet the criteria included in the SR design through the PICO elements. Thus, we intend to mimic this step by creating an ANN model.

Within PICO, only Population requires an understanding of the text, because there are several ways of describing a group of patients or samples. The terms generally used for Intervention, Control, and Outcome do not necessarily need a context, as they are often names of surgical techniques, medications, or scores. We believe that the search for the exact term will be appropriate.

Thus, the neural network will be automatically created with training on understanding Population. In each tested SR, the system will search PubMed for a MeSH (Medical Subject Headings) term (related to Population) for use as a database to develop an understanding of the text. The MeSH (Population) will be selected by the authors according to the design of each SR.

In addition to this database, we will add a MeSH search on the respective field to counteract and contextualize the creation of the neural network. Other fields, such as Intervention, Control, and Outcome, will be used in the form of tags to complement the article selection.

This specific model will be configured using the characteristics given in the following sections.

#### Network Configuration and Algorithm

The creation of the artificial neural network should be done through the Keras package, but there are other types of tools that we can use depending on the results found. The selection of scientific articles by reading the abstracts and title does not seem to require reading with semantic interpretation in which the word order is relevant, but rather with a selection of phrases that indicate a specific context. The word vectorization process is fundamental in the study design, impacting the final result of the model.

Thus, we will initially choose the *word2vec* and bag-of-words to capture a broad topical similarity through crosswords, but we will also test the Bidirectional Encoder Representations from Transformers [17]. The word classification will be done by multilayer perceptron (MLP) [18]. MLP is an analog of the artificial neural network, well known for its generalization and predictive capacity.

A neural network is created by stacking layers, and it is necessary to establish two main decisions in the model architecture. First, we will define how many layers will be required for the model. The second important decision to be made will be the number of hidden units used for each layer.

Regarding the model architecture, we will perform empirical adjustments of the hyperparameters during the execution. However, depending on the need, we can use some metaheuristics to optimize them, or even use an automated machine learning technique.

In addition, it will be essential to determine the parameters of the training algorithm and activation functions. This step will have a significant impact on the performance of the resulting system.

#### Loss Function and Optimizer

A model needs a loss function and an optimizer for training. The project in question will select the relevant and irrelevant articles for the outcome; thus, we can consider it a binary classification problem, and the model will generate a probability (a single-unit layer with sigmoid activation). The method used will be stochastic optimization by the Adam algorithm [19].

#### Model Training

Training will be controlled based on the model’s accuracy with out-of-sample data, creating a validation set by separating samples from the original training data. During training, model loss and accuracy will be monitored in the samples from the validation set.

In this step, we will adjust the weights of the connections, where it is essential to consider factors such as network initialization, training mode and training time. The training time has some variables that may influence the duration; therefore, a stopping criterion must be chosen, such as the average error rate per cycle or the generalizability of the network. Training will be interrupted when the network demonstrates an ideal generalizability and when an appropriate moment to stop with little error and maximum generalizability is found.

#### Model Testing

In this step, we will observe how the model behaves. This evaluation will be performed according to two returned values: the loss (a number representing the error) and the accuracy. The test set is used to determine the network’s performance with data not previously used. The network performance determined in the testing step will be a good measure of its actual performance. Other tests may also be used, such as network behavior analysis, through special inputs and analysis of current weights. If the values are very small, the connections can be considered insignificant and thus be eliminated.

### Comparison of Models

At the end of each model test, we will evaluate the model accuracy to assess whether the model contributed significantly to the final result. The model accuracy is defined as the measured difference between the final results of each SR reconstructed using manual selection. This assessment will determine whether it is necessary to use separate fields while searching for articles for the SR.

To elucidate this measurement, comparative analyses will be conducted for models created for the same field (intergroup comparison) and for two different fields (intragroup comparison). The receiver operating characteristic curve appears to be the best option for these analyses.

### System Sharing

The last step will be to share and test the system created for article selection in other fields. We will disseminate our project widely through the internet by creating the “Observatory of Evidence” in public health. In this location, other researchers with the same interest in improving the SR automation model will be able to include their searches, after internal validation, and thus expand and improve the created system. Authors should upload the results of their searches on the platforms, insert them into the system together with PICO, and choose the corresponding MeSH (Population).

The objective will be to publicize the observatory, promote the improvement of the automation system, and increase the capacity of the neural network model with more previously performed searches. In addition, this open platform will assist new studies in the field of public health, stimulating new systematic reviews.

## Results

Data collection will begin in July 2021. We estimate that data collection should be completed in December 2021, and the results should be available in March 2022.

## Discussion

Our aim is to disseminate scientific evidence through the “Observatory of Evidence” in public health that will be free and open to researchers. The authors will be able to upload the database returned from the search on the chosen platforms and add information, such as the respective field, types of studies to be selected by reading the abstracts, and inclusion criteria through the PICO strategy. After the researcher runs the model, the platform will return a list of suggested articles that meet predetermined criteria. The selection sensitivity will be modifiable.

This important tool for facilitating the creation of SRs will be made available through the platform. SR creation is often difficult because the large quantity of data involved can prevent potential reviews from being carried out. Most researchers perform article selection entirely manually or through reference management systems that only identify duplicate articles and do not perform abstract selection and reading.

Once the platform is operational, we intend to openly and freely disclose all the SRs generated through our automatic selection tool to all interested researchers. The system must routinely monitor performance and maintain the network when necessary. Some other improvements can be made based on researchers’ use of the SR automation, allowing the release of new versions with updates to better meet the needs of researchers.

The study has some limitations. The first and most important is that the system will depend on a corresponding MeSH or similar term for the study population for each systematic review. Another limitation is the use of three research databases (PubMed, Embase and Web of Science). Finally, the results will be studied for two areas of knowledge, limiting the generalization to other areas.

## Abbreviations

ANN: artificial neural networks
MeSH: Medical Subject Headings
MLP: multilayer perceptron
PICO: Population, Intervention, Control, and Outcome
PRISMA: Preferred Reporting Items for Systematic Reviews and Meta-Analyses
SR: systematic review
